# Comparison of the Field-Based Intermittent Running Fitness Test 30-15 and the Treadmill Multistage Incremental Test for the Assessment of Cardiorespiratory Fitness in Elite Handball Players

**DOI:** 10.3390/ijerph19063535

**Published:** 2022-03-16

**Authors:** Uros Mohoric, Marko Sibila, Ensar Abazovic, Sasa Jovanovic, Armin H. Paravlic

**Affiliations:** 1 Faculty of Sport, University of Ljubljana, 1000 Ljubljana, Slovenia; uros.mohoric@gmail.com (U.M.); marko.sibila@fsp.uni-lj.si (M.S.); 2 Faculty of Sport and Physical Education, University of Sarajevo, 71000 Sarajevo, Bosnia and Herzegovina; ensar.abazovic@fasto.unsa.ba; 3 Faculty of Physical Education and Sport, University of Banja Luka, 78000 Banja Luka, Bosnia and Herzegovina; sasa.jovanovic@ffvs.unibl.org; 4 Science and Research Centre Koper, Institute for Kinesiology Research, 6000 Koper, Slovenia; 5 Faculty of Sports Studies, Masaryk University, 625 00 Brno, Czech Republic

**Keywords:** VO_2max_, maximal running speed, aerobic endurance, aerobic incremental field test, laboratory treadmill test

## Abstract

The aim of the present study was to investigate whether the physiological parameters indicative of cardiorespiratory fitness obtained during the 30-15 intermittent fitness (30-15_IFT_) test and the multistage laboratory treadmill endurance (TR) test differ. Nineteen elite handball players were recruited for the current study and assigned in a cross-over manner to one of two tests to be performed 48 h apart at each visit to the testing facility. The results showed that VO_2max_ (percentage difference [PC] = 6.1%; *p* = 0.004) and maximal running velocity (V) (PC = 19.4%; *p* < 0.001) were significantly higher for the 30-15_IFT_ test than that obtained during the TR test. Furthermore, the onset of blood lactate accumulation was shown to be significantly higher for all measures considered to predict it during 30-15_IFT_ compared to TR as follows: VO_2max_ (PC = 12.6%; *p* = 0.001), running speed (PC = 33.9%; *p* < 0.001), and maximal heart rate (PC = 7.5%; *p* < 0.001). The current study highlights the importance of sport-specific testing, particularly for measuring individual cardiorespiratory fitness in elite handball players, as TR may underestimate crucial variables used for both diagnostics and training prescription.

## 1. Introduction

Handball, which has been known in its current form since 1954, has developed rapidly in recent decades as an Olympic and professional sport. Handball is a game characterized by frequent role changes of teams in attack and defense [1], with specific movement patterns consisting of different types of fast and explosive muscle actions, such as jumping, forward and backward sprinting, turning, and various changes in direction, with constant changes in tempo in combination with different types of ball throwing [2,3]. The characteristic movement patterns of the players, which are repeated in the different phases of the game, have different effects on their physiological load [4,5]. In addition to anthropometric measures, ball throwing speed, and technical and tactical skills, studies have shown that players’ success depends in part on their ability to repeat short high-intensity runs [4,6]. Moreover, handball players have been shown to have a heart rate greater than 80% of their individual maximum heart rate (HR_max_) during 50% of their total effective playing time [5], suggesting that cardiorespiratory fitness (CRF) is one of the most important determinants of performance in handball.

Strength and conditioning professionals in handball must be able to administer time-efficient, valid, and reliable tests to evaluate the various fitness capacities of their players. Indeed, direct measurement of CRF during a multistage laboratory treadmill endurance test (TR) is considered the “gold standard” for estimating maximal oxygen uptake (VO_2max_) [7]. Although not handball specific, TR is still routinely used to assess CRF in handball players [6,8,9]. However, due to its high cost, complex measurement procedures, and inability to measure more than one athlete at a time, this method is not the most appropriate for most handball teams. In addition, various blood lactate indices have been proposed to measure athletes’ ability to exercise without accumulating lactate [10]. For example, onset of blood lactate accumulation (OBLA) was found to be strongly correlated with endurance performance (*r* = 0.96) [11] and has been proposed as a significant predictor of running performance [12], sensitive enough to discriminate between elite and non-elite athletes [13]. Therefore, it is important for coaches to determine the running intensity at which OBLA occurs to monitor CRF performance and prescribe endurance training intensities to their athletes. However, the point at which OBLA occurs may vary when different types of exercise [14] and testing are considered [15].

In practice, various laboratory and field tests are used to determine the physiological responses of athletes to endurance exercise. The advantage of field testing is that a larger number of athletes can be assessed at the same time, which saves time and other resources. Field tests have emerged that are better adapted to the specific requirements of various sports than laboratory tests [9,16]. They allow more suitable assessment of CRF that can be more specific to the actual sport in question [17,18]. In this way, field tests have evolved in different directions in terms of modality, i.e., the continuous test (the University of Montreal track test [19]), and/or the intermittent multistage fitness test [20] or Yo-Yo test. In these tests, it is often the case that athletes with a lower maximal running velocity (V) run at higher levels of intensity just to maintain the pace of athletes with a higher V [21]. Consequently, using the Yo-Yo test to prescribe a training intensity would not be a best option [21]. The desire to solve this problem, while also improving interval training prescription in team sports, led to the development of the 30-15 Intermittent Fitness Test (30-15_IFT_) [22]. The 30-15_IFT_ consists of 30 s shuttle runs interspersed with 15 s active recovery periods. At the beginning of the test, a running speed is set at 8 km/h for the first 30 s run and increased by 0.5 km/h in each 30 s phase thereafter. Subjects are required to run back and forth between two lines 40 m apart at the preset pace determined by a pre-recorded beep. The speed of the last successfully completed stage is recorded as the test result, i.e., the maximum running speed (V) during 30-15_IFT_ (V_IFT_) [23]. By implementing this basic idea, the 30-15_IFT_ is useful for assessment of cardiorespiratory fitness by providing accurate estimation of maximal oxygen uptake (VO_2max_), HR_max_, and other components relevant to sports-related performance and training optimization, such as maximal aerobic speed and intermittent running effort recovery capacity [22]. Buchheit [16] showed that when using continuous CRF tests, running speed at maximal oxygen uptake is significantly lower than running speed at 30-15_IFT_, suggesting that metabolic load at 30-15_IFT_ is much closer to loads observed during a handball match. Thus, compared to TR tests, the 30-15_IFT_ seems to be more specific to handball match demands [9]. In addition, V_IFT_ can be considered to be an optimal tool for individualizing short intermittent run distances in handball players [23].

The first application and validation of the 30-15_IFT_ was performed on a sample of handball players [16]. With time and the popularization of the test, it was also used in other sports, with 30-15_IFT_ validations conducted in basketball, field hockey, football, and rugby [16]. The mentioned studies also confirmed the high reliability of different parameters estimated from the 30-15_IFT_ (ICC = 0.90–0.96). In practice, studies comparing the 30-15_IFT_ with other tests (YO-YO IR1, YO-YO IR2, 20 m shuttle run test) are becoming more common to determine its validity and potential for use in different sports [16,23,24].

To date, the concurrent validity of the 30-15_IFT_ has not been investigated in elite handball players compared with the standard continuous incremental running test. Moreover, the relationship between VO_2max_ measured with a portable metabolic measurement system (VO_2maxIFTK4_) and the predicted value (VO_2maxIFT_) has yet to be determined. Therefore, the aim of this study was to experimentally test whether the 30-15_IFT_ can be used as valid indicator of CRF and HR_max_. We also wanted to investigate the differences between the 30-15_IFT_ and the TR test considering a point at which OBLA occurs. This information may help coaches determine CRF and prescribe endurance training intensities for their athletes when considering different testing protocols.

## 2. Materials and Methods

### 2.1. The Experimental Approach to the Problem

All handball players performed two maximal exercise tests, one on the field (IFT_30-15_) and one in the laboratory (i.e., TR), to evaluate their cardiorespiratory fitness in the middle of the regular training season. During this period, athletes performed mainly tactical and technical training with handball game-specific movement patterns and physiological efforts (4 to 7 sessions), while strength and power training was performed once or twice a week. During the first visit to the testing facility, the TR was performed indoors on a treadmill, whereas the field test was performed on a standard indoor handball court with a hardwood floor. The tests were conducted with a rest period of 48 h between them. Both tests were conducted between 10 am and 11 am at an ambient temperature of 20 to 22 °C.

### 2.2. Participants

During the conceptualization of the study design, an a priory power analysis was conducted based on the correlation coefficient, as recommended [25]. Based on a previous study with a similar aim and design [22], we expected to find a moderate to high relationship between continuous and intermittent run tests (r = 0.76) for VO_2max_. Therefore, with a probability of failing to reject the null hypothesis of β = 0.2 and two-tailed α = 0.05, a minimum sample size of 11 subjects was shown to be sufficient to detect a value of ≥0.76 for r. As a result, twenty-four elite handball players (mean age: 24.2 ± 5.5 years; height 188.6 ± 6.5 cm; body mass 89.4 ± 9.4 kg; training status 12.6 ± 3.1 years) were recruited for the current study. The inclusion criteria were as follows: handball players who are members of a national team and/or play at the international level and regularly participate in European Cups, who have not had any serious injury or illness that could limit maximal performance for the six months prior to the start of the study, who have not had acute pain, and who have participated in a regular training process. To avoid unnecessary fatigue, players and coaches were instructed to avoid intense sporting activities one week before actual testing and during the study period. All subjects were informed of the benefits and potential risks of the study and provided written informed consent to participate in the current study. All procedures were conducted in accordance with the ethical standards of the 1964 Declaration of Helsinki and approved by the Ethics Committee of the Faculty of Sport (University of Ljubljana, Ljubljana, Slovenia).

### 2.3. Procedures

Immediately prior to testing, participants completed a standard 25 min warm-up program consisting of 10 min of self-paced jogging, 10 min of dynamic stretching, and 5 repetitions of 30 m of fast running. After the warm-up routine participants each participant performed the TR or IFT test.

### 2.4. Field Test

Aerobic capacity was measured using the field based 30-15_IFT_ test, as previously recommended [23,26]. This intermittent, incremental test consists of 30 s shuttle runs interspersed with 15 s active recovery periods. Running speed was set at 8 km/h for the first 30 s run and increased by 0.5 km/h in each 30-s phase thereafter. Players were required to run back and forth between two lines 40 m apart at the preset pace determined by a pre-recorded beep. The prerecorded beep allowed players to adjust their running speed when they entered a 3 m zone in the middle and at each end of the test field. During the 15 s recovery period, players walked forward to the nearest line (either in the middle or at the end of the running area, depending on where their previous run had ended); from this line, they began the next running phase. Players were instructed to complete as many stages as possible. The test ended when the player could no longer maintain the required running speed or failed to reach the 3 m zone three consecutive times in the period before the sound signal. The speed of the last successfully completed stage was recorded as the test result, i.e., the maximum running speed (V) during 30-15_IFT_ (*V_IFT_*) [23]. The *VO_*2*max_* was calculated by following equation [23]:

$$VO_{2maxIFT}\;\left(\frac{ml}{\frac{min}{kg}}\right)=28.3-2.15G-0.741A-0.0357BM+0.058A\times V_{IFT}+1.03V_{IFT}$$

where (*G*) stands for gender, (*A*) for age and (*BM*) for subjects’ body mass.

### 2.5. Incremental Treadmill Test

An incremental step test was performed on a treadmill (hp Cosmos Saturn, hp Cosmos, Traunstein, Germany) with a constant gradient of 1% inclination [27]. The initial velocity was set at 8 km/h and increased by 2 km/h every 4th minute until the subject was no longer able to maintain the velocity. The achievement of VO_2max_ was identified as the plateauing of VO_2_ (<2.1 mL/kg/min decrease) despite an increase in workload [28]. If the above-stated criterion was not fulfilled, the participants were asked to perform a further constant-speed test equal or higher than the highest speed achieved at the end of the incremental test, as recommended [29]. Throughout the test, respiratory gases were continuously measured breath-by-breath and reduced to 10 s averages [30]. The rest period between different stages was 1 min, which was used to take lactate samples. The last running velocity reached during the test was defined as V_TR._

### 2.6. Maximum Aerobic Performance, Heart Rate and Blood Sampling Testing Equipment

A portable gas analyzer K4b^2^ (COSMED, Rome, Italy) was used to obtain physiological parameters. The device provides reliable values for oxygen uptake (O_2_), carbon dioxide production (CO2), and pulmonary ventilation (VE) breath-by-breath [31,32]. In addition, blood samples (20 μL) were collected from the earlobe for both tests and the samples were analyzed for blood lactate concentration (LA-) using a Kodak EKTACHROME analyzer. However, due to the different natures of the tests performed, blood samples were collected at the following intervals:

30-15_IFT_ test: before the test (T1); at running speeds of 9 km/h (T2), 10.5 km/h (T3), 12 km/h (T3), 13.5 km/h (T4), 15 km/h (T5), 16.5 km/h (T6), 18 km/h (T7), 19.5 km/h (T8), 21 km/h (T9), and 22.5 km/h (T10); and at 3rd (T11) and 5th (T12) minutes after the end of the test.

TR test: before the test (T1); at running speeds of 10 km/h (T2), 12 km/h (T3), 14 km/h (T3), 16 km/h (T4), and 18 km/h (T5); and at 3rd (T6) and 5th (T7) minutes after the end of the test.

Simultaneously, a heart rate was measured by Polar S-610 heart rate pulse-meters (Polar Electro, Kempele, Finland). The data were recorded in 5 s intervals and automatically analyzed using the original Polar software. The OBLA that is the running speed corresponding to the [LA] of 4 mmol/L was detected by interpolation from the [LA]–running speed relationship curve [33].

### 2.7. Statistical Analysis

All data are presented as mean ± SD and 95% confidence intervals. Statistical analyses were undertaken with SPSS statistical software (version 27, IBM corp., Chicago, IL, USA). Normality was confirmed by visual inspection and using the Shapiro–Wilk test. The paired Student’s *t* test was used to compare physiological data obtained by laboratory and field-based tests. One-way ANOVA was used to compare the VO_2max_ values obtained on TR (VO_2maxTR_), VO_2maxIFTK4_, and VO_2maxIFT_. Hedges’ g effect sizes (ES) with 95% confidence intervals were calculated to show practical differences between legs and were interpreted as: trivial: <0.20, small: 0.20–0.50, moderate: 0.50–0.80, or large: >0.80 [34]. Bland–Altman analysis was used to determine absolute limits of agreement between (a) VO_2maxTR_ and VO_2maxIFTK4_; (b) VO_2maxIFTK4_ and VO_2maxIFT_; and (c) V_TR_ and V_IFT_. In addition, a Pearson correlation coefficient was used to evaluate the association between VO_2max_ variables obtained from TR and IFT tests, whereas Spearman’s rank-order correlation was used if the assumption of normality of data distribution was violated. The following thresholds of the correlation coefficient were used to assess the magnitude of the relationships analyzed: weak ≤ 0.35; 0.36 ≤ moderate < 0.67; 0.68 ≤ high < 1 [35]. A level of significance for all analyses was accepted at *p* ≤ 0.05.

## 3. Results

Nineteen players (five backcourts, seven wings, and seven line/pivots) completed both testing protocols and were included in a final analysis. Table 1 shows the comparison of the main physiological parameters obtained during TR and 30-15_IFT_ tests. The results showed that VO_2max_ (percentage difference [PC] = 6.1%; *t* = 3.342; *p* = 0.004) and maximal running velocity (PC = 19.4%; *t* = 12.669; *p* < 0.001) were significantly higher for 30-15_IFT_ than that obtained during TR test. Moreover, OBLA was shown to be significantly higher for all measures considered to predict its onset during 30-15_IFT_ compared to TR as follows: VO_2max_ (PC = 12.6%; *t* = 4.421; *p* = 0.001), running velocity (PC = 33.9%; *t* = 15.484; *p* < 0.001), heart rate (PC = 7.5%; t = 6.348; *p* < 0.001), and respiratory exchange ratio (PC = 7%; *t* = 3.372; *p* = 0.003).

**Figure 1:**
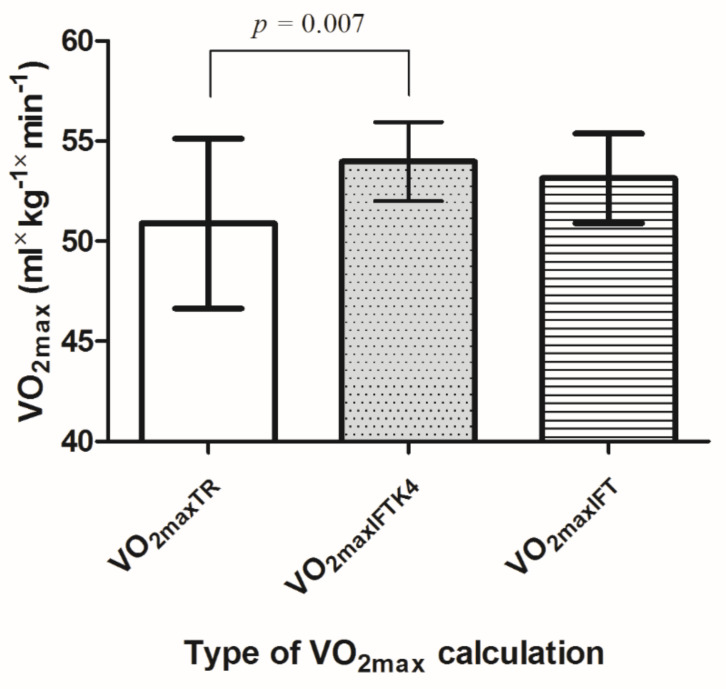
Comparison of VO_2max_ values obtained by the continuous treadmill test (VO_2maxTR_), 30-15_IFT_ measured by a portable metabolic analyzer (VO_2maxIFTK4_), and the 30-15_IFT_ proposed calculation (VO_2maxIFT_).

Figure 1 indicates a significant difference between VO_2maxTR_, VO_2maxIFTK4_, and VO_2maxIFT_ (F_2_ = 5.398; *p* = 0.007). Post hoc comparison showed that VO_2max_ was significantly lower for TR than that obtained during 30-15_IFT_ measured with a gas analyzer i.e., IFT_K4_ (PC = 6.1%, *p* = 0.007), but had only a trend towards significance when compared to the predicted value from the IFT result (PC = 4.4%, *p* = 0.074).

**Figure 2:**
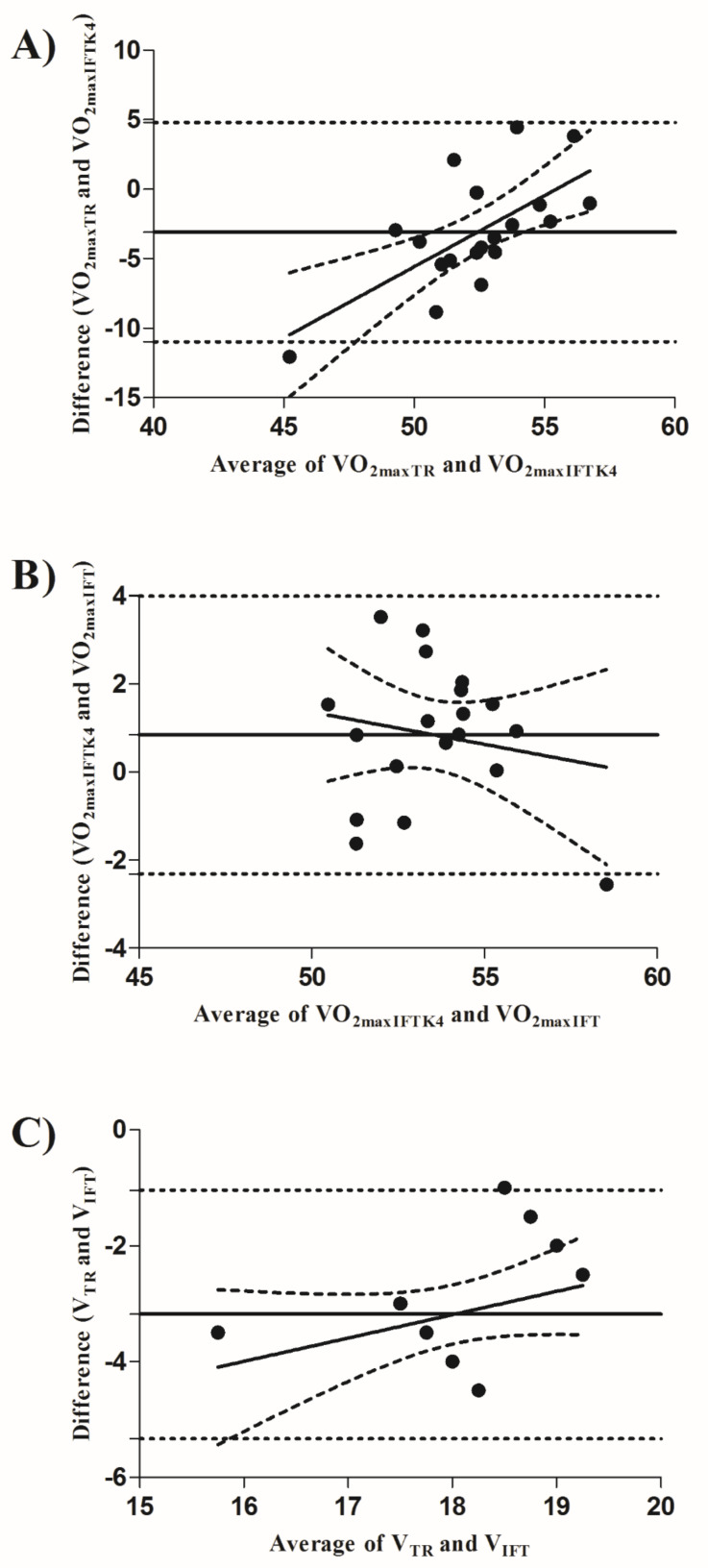
Bland–Altman plots comparing VO_2maxTR_ and VO_2maxIFTK4_ (**A**); VO_2maxIFTK4_ and VO_2maxIFT_ (**B**); V_TR_ and V_IFT_ (**C**).

In addition, the variations in the differences between the two tests were significant and did not fell within the limits of agreement for VO_2maxTR_ and VO_2maxIFTK4_ (Figure 2A; LOA 95% = −10.97 to 4.80), VO_2maxIFTK4_ and VO_2maxIFT_ (Figure 2B; LOA 95% = −2.31 to 3.99), and V_TR_ and V_IFT_ (Figure 2C; LOA 95% = −5.33 to −1.04). Furthermore, significant positive correlations between VO_2maxTR_ and VO_2maxIFT_ (moderate *r* = 0.512, *p* = 0.025), and VO_2maxIFTK4_ and VO_2maxIFT_ (high *r* = 0.715, *p* = 0.001) were found, whereas correlations between V_TR_ and V_IFT_ (weak *r_s_* = 0.314, *p* = 0.191), and VO_2maxTR_ and VO_2maxIFTK4_ were not significant (weak *r* = 0.339, *p* = 0.155).

## 4. Discussion

The primary aim of the study was to compare physiological parameters obtained during TR and 30-15_IFT_ tests in elite handball players. In addition, a concurrent validity and correlation analysis was performed between determined VO_2max_ and V values. Considering the characteristics of handball [1] and the principles of training specificity and adaptation, we hypothesized that the 30-15_IFT_ test provides better insight into the physiological parameters of handball players than the traditional TR test.

The results showed that the values for V and VO_2max_ were significantly higher in 30-15_IFT_ than in the TR test. V values obtained are consistent with previously reported results, showing between 2 and 5 km/h higher running speed during 30-15_IFT_ than those achieved during the TR test [16]. Later results suggest that the 30-15_IFT_ is a sensitive tool for detecting changes in V during performance monitoring. In addition, similar trends in VO_2max_ difference estimates have been reported for female soccer players [36], female basketball players [37], and male team sports athletes [22]. There are two main reasons that may explain the discrepancies observed. First and foremost, the 30-15_IFT_ is a field test that assesses change in direction ability, inter-effort recovery ability, and anaerobic capacity in addition to CRF [16], and is more similar to the handball game than a TR test, which uses a continuous running protocol. Furthermore, group testing, as performed in team sports during the 30-15_IFT_ test, has been shown to increase task motivation [38]. In addition, our results demonstrated that HR_max_ values tend to be higher during 30-15_IFT_, confirming previous findings [22,36,37], although with lack of statistical significance. This is not surprising, because sport-specific tests often result in higher HR_max_ values, as shown in other sports such as soccer, tennis, and squash [39,40,41].

When comparing OBLA, the estimated values differ significantly between TR and 30-15_IFT_. OBLA was found to be significantly higher for all measures used to predict it during 30-15_IFT_ compared with TR. The observed differences can be explained by the protocols used, which comprise intermittent versus continuous endurance testing. The unique feature of 30-15_IFT_ is that the running bouts are interrupted by a 15 s active rest, which enables athletes to partially resynthesize the energy substrates used for both the anaerobic (i.e., ATP and CP) and aerobic components of intermittent running performance (i.e., myoglobin functioning) [42]. Essen and colleagues showed that ATP and CP levels fluctuated between work and rest phases during the intermittent running protocol, but did not resynthesize to initial levels. In addition, myoglobin functioning, which acts as an oxygen store, was found to be an important factor in delaying OBLA, the time when anaerobic metabolism becomes dominant during maximal endurance testing. It appears that a 15 s rest period can delay the onset of fatigue, allowing athletes to achieve a 19.4% higher V during 30-15_IFT_ compared to TR.

To the best of the authors’ knowledge, this is the first study to report objectively measured and estimated 30-15_IFT_ VO_2max_ values. Although several studies have shown that 30-15_IFT_ is reliable, valid, and useful [36,43], no author has compared the objectively measured VO_2max_ values and those calculated according to the formula proposed by Buchheit [23].

We found moderate and high correlations between 30-15_IFT_ and TR and 30-15_IFT_ and 30-15_IFT4K_ VO_2max_ values, respectively, whereas 30-15_IFT4K_ and TR values had a weak, non-significant correlation. These results confirm our hypothesis that the 30-15_IFT_ test can provide better insight into the physiological parameters of handball players than the traditional TR test, due to its similarity to handball play. This was further supported by the significant difference between TR and the 30-15_IFT_ VO_2max_ values, which underestimated VO_2max_ by up to 6.1% during the continuous endurance test.

This study highlights the importance of sport-specific testing, particularly for measuring individual CRF. As noted by Basset and Boulay [44], VO_2max_ is highly dependent on the type of test. For example, runners are usually tested on a treadmill and cyclists on a cycle ergometer because of this specific adaptation. The 30-15_IFT_ test takes into account various qualities required in shuttle intermittent runs, such as lower limb power, aerobic qualities, and the ability to recover between subsequent sets of running bouts [16], and plays a very important role in diagnostics, training prescription, and optimization in handball.

We are aware that our study may have some limitations. Although we used a reliable and valid multistage laboratory testing protocol to assess CRF, it may not be the most appropriate protocol to compare physiological strain at different levels of running intensity with 30-15_IFT._

## 5. Conclusions

We found that the variations in the differences between two graded treadmills and IFT were significant and did not fall within the limits of agreement. In addition, the results showed that the values for maximum running velocity and VO_2max_ were significantly higher in the 30-15_IFT_ test than in the TR test. Therefore, the current study highlights the importance of sport-specific testing, particularly for measuring individual cardiorespiratory fitness in elite handball players, as TR may underestimate crucial variables used for both diagnosis and training prescription.

## Figures and Tables

**Table 1:** Comparison of main physiological parameters obtained during continuous treadmill test and 30-15_IFT_ test.

	Treadmill Test		30-15_IFT_ Test								
	Mean	SD	Mean	SD	∆	PC %	Hedges’ g	LLCI	ULCI	t Value	*p* Value
VO_2max_ (mL/kg/min)	50.89	4.24	53.98	1.97	3.09	6.1	0.75	(0.24 to 1.25)		3.342	0.004
Maximal running speed (km/h)	16.42	1.26	19.61	0.92	3.18	19.4	2.85	(1.82 to 3.86)		12.669	0.000
Maximal heart rate (bpm)	183.95	8.46	184.84	9.03	0.89	0.5	0.20	(−0.25 to 0.64)		0.892	0.384
Respiratory exchange ratio	1.07	0.20	1.05	0.16	−0.03	−2.3	−0.20	(−0.64 to 0.25)		−0.900	0.380
Maximal lactate during test (mmol/L)	10.14	3.63	8.94	3.12	−1.21	−11.9	−0.26	(−0.71 to 0.19)		−1.174	0.256
Lactate at 3 min after test	10.24	2.76	8.61	3.05	−1.63	−16.0	−0.53	(−1.00 to -0.03)		−2.282	0.036
Lactate at 5 min after test	9.73	3.20	8.41	3.27	−1.33	−13.6	−0.40	(−0.86 to 0.08)		−1.724	0.103
OBLA based on VO_2max_ (mL/kg/min)	42.16	4.83	47.45	5.63	5.30	12.6	0.95	(0.40 to 1.47)		4.210	0.001
OBLA based on running speed (km/h)	12.95	1.34	17.34	1.35	4.39	33.9	3.48	(2.26 to 4.68)		15.484	0.000
OBLA based on HR (bpm)	163.89	11.40	176.26	8.96	12.37	7.5	1.43	(0.78 to 2.05)		6.348	0.000
OBLA based on RER	0.90	0.12	0.97	0.10	0.06	7.0	0.76	(0.25 to 1.25)		3.372	0.003
HR at 3 min after test (bpm)	121.26	13.11	118.63	14.55	−2.63	−2.2	−0.18	(−0.62 to 0.27)		−0.780	0.446
HR at 5 min after test (bpm)	107.47	10.84	109.63	11.70	2.16	2.0	0.20	(−0.25 to 0.64)		0.874	0.394

∆—difference in means between continuous VO_2max_ and 30-15_IFT_ tests; PC—percentage difference; LLCI—lower limit confidence interval; ULCI—upper limit confidence interval; OBLA—onset of blood lactate accumulation; HR—heart rate; bpm—beats per minute; RER—respiratory exchange ratio.

## Data Availability

All data generated are available within the present manuscript.
